# The Relationship Between the Customer Relationship Management and Patients’ Loyalty to Hospitals

**DOI:** 10.5539/gjhs.v8n3p65

**Published:** 2015-06-25

**Authors:** Shadi Hajikhani, Seyed Jamaledin Tabibi, Leila Riahi

**Affiliations:** 1 Department of Health care Services Management, Faculty of Medical Sciences, Science and Research Branch of Tehran, Islamic Azad University, Tehran, Iran

**Keywords:** CRM, organization and management, patients, hospitals

## Abstract

**Background and Aim:**

Customer Relationship Management (CRM) with its various components has been considered as a tool causing customers’ loyalty. The present study aims to investigate the relationship between the various components of customer relationship management and patients’ loyalty to the place of their treatment.

**Methods:**

This cross sectional and descriptive-analytical study was conducted among nurses and hospitalized patients in inpatient wards in selected hospitals in 2014. Using the stratified random sampling method, 224 valid and reliable researcher-drafted questionnaires were completed for CRM by nurses and 359 questionnaires were completed by patients for patients’ loyalty in the studied wards. Data were analyzed using the SPSS_20_ software.

**Results:**

There was no statistically significant relationship between the level of patients’ loyalty and organizational indicators, information technology and knowledge management (P Value>0.05). However, there was a statistically significant relationship between loyalty and the dimensions of the service process (P Value=0.04), human resources (P Value=0.002) and CRM (P Value=0.038). The strength of these relationships were 34, 40 and 36 percent, respectively all of which were positive.

**Conclusion:**

Customer Relationship Management is a tool for improving influencing factors on patients’ satisfaction and loyalty. Therefore, attempts to implement customer relationship management as a process for improving hospitals performance and improving communication between service providers in hospitals and customers leading to enhance patients’ loyalty should be taken into account by managers and policy makers in the health sectors.

## 1. Background

Attracting and maintaining customers have long been believed to be of main aspects of activities in every organization. New forms of competition and structural changes in transactional processes have led to the creation of patterns for long term communication between stakeholders in market (Šebjan, Bobek, & Tominc, 2014). However, the competition has increased in various businesses due to the diversification of customers’ demands. Indeed, many businesses try to build effective communication with customers in order to consider their needs. In addition, they found out that maintaining current customers is cheaper than attracting new ones. To do so, Customer Relationship Management (CRM) systems have been implemented in many cases (Choi et al., 2013; Lee, 2012; Tohidi & Jabbari, 2012). Thus, attracting and maintaining customers and creating loyalty among them in organizations are considered as aims of the establishment of the CRM (Ngai, 2005).

Is said that CRM, as a new term, was widespread since the mid-1990s (Choi et al., 2013; Ngai, 2005). This system is a marketing technique and an investment approach through which an organization can understand customers and influence customers’ behavior. All this is done through providing customized services for each group of customers and a significant relationship in order to acquire more customers, retain customer, increase customers’ loyalty and improve customers’ profitability (Choi et al., 2013; King & Burgess, 2008; Kostojohn, Johnson, Paulen, & McKinnie, 2011; Lee, 2012).

There are several factors influencing customers’ loyalty such as employee satisfaction, employee loyalty and the quality of services (Arab, Tabatabaei, Rashidian, Forushani, & Zarei, 2012; Caruana, 2002; Tabibi, Kakhani, Gohari, & Shahri, 2009).

Nowadays, the number of health care centers has increased along with the increased competition in the health care industry. This has resulted in establishing the customer relationship management system in order to meet diverse demands of customers. Due to the intensifying competition among health care centers, services tailored to customers are provided through analyzing their needs by the centers (Choi et al., 2013; Trepper, 2000).

The CRM for health care service providers is an approach through which they learn whatever related to their patients, their viewpoints and expectations in order to make relationship with them, provide timely information and follow up their related results so that they can carry out corrective measures, increase customer loyalty and gain more profit (S.-Y. Hung, W.-H. Hung, Tsai, & Jiang, 2010). Also, CRM is a way to maximize patients’ satisfaction through identifying patients’ needs and providing high quality health care services based on patients’ preferences (Choi et al., 2013).

A favorable relationship between health care providers and patients not only enhances patients’ satisfaction but also improves the quality of health (Almunawar & Anshari, 2012; Beak, 2008). The importance of this issue is to the extent that issues related to CRM such as the provision of care to uninsured individuals, medical/hospital interactions and customers’ satisfaction have been categorized among 9 high priority factors in hospitals (Hung et al., 2010).

## 2. Objective

Most of the past attempts by hospitals related to the implementation of the CRM have been wrongly directed and supported (Young, 2007). It is therefore necessary to investigate and prioritize affairs required for establishing and implementing CRM. This study aims to investigate the relationship between the dimensions of CRM in hospital wards and patients’ loyalty in order to help managers identify influential dimensions of this technique on patients and their loyalty.

## 3. Methods

This cross sectional and descriptive-analytical study conducted in 2014 aimed to investigate the relationship between customer relationship management and patients’ loyalty. The study was conducted in 34 inpatient wards in selected hospitals affiliated to Tehran University of medical sciences. Nurses (N=429) and patients (N=4127) in inpatient wards in the studied hospitals were the population of this study. In order to determine the sample size, at first the Morgan's table and stratified random sampling method were used and the total number of sample for all hospitals were calculated (S=583) (S _Nurse_= 224 & S _Patients_=359). In each hospital, the number of nurses and patients were determined in terms of wards and this number of sample size proportional to the number of nurses and patients was distributed.

Two questionnaires including the CRM questionnaire and the patient loyalty questionnaire were used to gather data. The CRM questionnaire was a researcher-drafted one consisting of two parts. The first part included demographic information of participants and the second part was consisted of 42 questions in 5 areas - organizational indicators (9 questions), service-providing process (9 questions), human resources (12 questions), information technology (6 questions) and knowledge management (6 questions). This questionnaire was designed based on the 5-point Likert scale consisting of very high (5 point), high (4 point), moderately (3 point), low (2 point) and very low (1 point). The validity of the questionnaire which was based on the collective judgment of scholars and experts and by using the content validity index (CVI) was calculated to be 84%. Also, in order to assess the reliability of the questionnaire, the Cronbach's alpha was calculated to be 96%. As to measure patients’ loyalty, the questionnaire used by Tabibi et al. (Tabibi et al., 2009) was used which consisted of 5 questions; that validity and reliability the Cronbach's alpha was calculated to be 86%.

The researcher distributed the CRM questionnaire among nurses working in morning, evening and night shifts in 34 inpatient wards in the studied hospitals. Also, the patients’ loyalty questionnaire was distributed among the hospitalized patients at the point of discharge in these 34 wards.

The SPSS 20 software and descriptive and inferential statistical methods were used to analyze data. Descriptive statistics were used to draw the frequency distribution tables and inferential statistics were used to determine differences and the relationship between variables. The Kolmogorov-Smirnov test (KS-test) was used to assess the normality of the scores distribution. Also, Pearson's and Spearman's correlation test were performed to assess the correlation between variables. To address ethical and legal issues, a letter of permission was issued by the relevant university. Also, the participants were given sufficient explanations and they were assured that the questionnaires information will be kept confidential.

## 4. Results

Among the nurses, 96% were female, 50% were married and 96% had a Bachelor of Science degree in nursing; 88% were nursing experts and 9% were head nurses. The average age of the nurses was 30.54±5.1 years and the average years of work experience among them was 6.8±4.8 years. Among the patients, 52% were female, 85% were married, 18% had college degrees, 43% were housewives, 41% were salaried employees in the public and private sectors, 62% were over the age of 40. Among the 93% of those covered by the health insurance, 73% had basic health insurance and 27% of them were covered by complementary health insurance, besides the basic health insurance.

Among the 34 studied wards, the mean score of loyalty was 3.43; the mean score of organizational indicators was 3.39; the mean score of service process was 3.48; the mean score of human resources was 3.77; the mean score of information technology was 3.44; the mean score of the knowledge management was 3.34 and the mean score of CRM was 3.52. The highest attainable score was 5.

The Kolmogorov-Smirnov test showed that all the scores of loyalty, organizational indicators, service- providing process, human resources, information technology, knowledge management and the customer relationship management were normally distributed.

**Table 1:** Results of Kolmogorov-Smirnov normality test for the variables of loyalty and CRM and its 5 areas in the studied hospitals

Variable	Kolmogorov- Smirnov			Mean

	Statistics	Df	Sig.	
**Loyalty**	0.07	34	0.2	3.43
**Organizational indicators**	0.08	34	0.2	3.39
**Service providing process**	0.09	34	0.2	3.48
**Human resources**	0.10	34	0.2	3.77
**Information technology**	0.09	34	0.2	3.44
**Knowledge management**	0.10	34	0.2	3.34
**CRM**	0.10	34	0.2	3.52

The findings of Table 2 indicates that there was no statistically significant relationship between the level of patients’ loyalty and organizational indicators, information technology and knowledge management (P Value>0.05). However, there was a statistically significant relationship between loyalty and the dimensions of the service-providing process (P Value=0.04), human resources (P Value=0.002) and the CRM (P Value=0.038).

**Table 2:** The correlation between the customer relationship management and its components and patients’ loyalty in the studied hospitals

		Loyalty	Organizational Indicators	Service- Providing Process	Human Resources	Information Technology	Knowledge Management	CRM
**Loyalty**	Pearson's correlation	1	0.3	0.34*	0.40*	0.2	0.3	0.36*
	decision criterion (2-tailed)		0.1	0.04	0.002	0.2	0.1	0.038
**Organizational indicators**	Pearson's correlation		1	0.81**	0.6	0.7	0.8	0.9
	decision criterion (2-tailed)			0.001	0.006	0.001	0.008	0.003
**Service- providing process**	Pearson's correlation			1	0.81**	0.6	0.7	0.9
	decision criterion (2-tailed)				0.004	0.003	0.001	0.001
**Human resources**	Pearson's correlation				1	0.84**	0.63**	0.86**
	decision criterion (2-tailed)					0.003	0.002	0.001
**Information technology**	Pearson's correlation					1	0.776**	0.877**
	decision criterion (2-tailed)						0.004	0.002
**Knowledge management**	Pearson's correlation						1	0.889**
	decision criterion (2-tailed)							0.001
**CRM**	Pearson's correlation							1
	decision criterion (2-tailed)							-

* Correlation is significant at the 0.05 level (2-tailed);

** Correlation is significant at the 0.01 level (2-tailed).

The assessment of the correlation between the CRM components with each other and with CRM also indicates that there is also a significant relationship between all these components. This is while the dimensions of organizational indicators and the service-providing process showed the highest correlation with CRM (90 percent) and the dimension of human resources showed the lowest correlation with CRM (86 percent).

## 5. Discussion

The results of the study showed that there is a statistically significant difference between the level of loyalty among male and female in the studied population (P Value=0.02). However, there is no statistically significant correlation between demographic features including marital status, age, the status of insurance and the type of insurance, the level of education and the status of employment with patients’ loyalty. This issue indicates that the perception among patients in various groups in the studied population is nearly the same.

There is no statistically significant relationship between the level of patients’ loyalty and organizational indicators, information technology and knowledge management. However, there is a statistically significant and positive relationship between loyalty and the dimensions of the service-providing process, human resources and the CRM. There is also significant relationship between CRM and all its components; while the dimensions of organizational indicators and the service-providing process showed the highest correlation with the CRM. In other words, there is a significant relationship between customer relationship management and patients’ loyalty, however, no relationship was observed in the areas of organizational indicators, information technology and knowledge management. It may be concluded that patients can well understand the two areas of human resources and service-providing process and express their opinion in this regard. However, the areas of organizational indicators, information technology and knowledge management are not observable and understandable for patients.

There are strong correlations between customer relationship management and its dimensions (organizational indicators, the service-providing process, human resources, information technology and knowledge management).

Consistent with these results, Foss also regarded the cooperation among various sections, as one of the components of organizational indicators, as a necessary approach for customer relationship management (Foss, Stone, & Ekinci, 2008). Dewhurst et al. stated in their study that information technology has facilitated and increased the relationship with customers in various ways and has enabled organizations for personalization (Dewhurst, Martínez Lorente, & Dale, 1999). In a study on success factors in customer relationship management in hospitals conducted by Hung et al. considered the existence of knowledge management in hospitals as a necessary component for the success of CRM (Hung et al., 2010). In this regard, Hussain et al. also acknowledged the importance of human resources and the way of providing services (Hussain et al., 2014).

The results of the Pearson's correlation test show that there is a significant correlation between customer relationship management and patients’ loyalty to the medical ward.

Cobelas et al. concluded that when a hospital meets a patient's needs at a higher level than he/she expects, then this patient will not change the hospital (Cobelas et al., 2001). Adeleke (Adeleke & Aminu, 2012) introduced three factors of ‘the quality of services’, ‘customer satisfaction’ and ‘the perception of participation’ as three main factors of customer loyalty. The customer relationship management is a tool creating these three factors, so these findings are consistent with those of this study.

Also, Galbreath and Rogers have considered ‘customization’, ‘customized communication’ (personalization for the individual customer) and ‘providing after sales support services’ as the three main goals of customer relationship management (Galbreath & Rogers, 1999). Paazine stated that the improvement in customer services, cost reduction as well as customer retention and loyalty are the basic aims of implementing CRM in hospitals (Paazine, 2011). The results of this study are also consistent with those of the current study. Furthermore, in a study conducted by Gbadeyan, stated that CRM has had a significant impact on the quality of hospital services in Nigeria which in turn creates patient satisfaction and loyalty (Gbadeyan, 2010).

A significant relationship between the component of service-providing processes and loyalty was observed. Arab et al. have reported the process quality as one of the dimensions influencing loyalty (Arab et al., 2012). Kessler and Mylod (Kessler & Mylod, 2011) also stated that the process of service provision plays an important role in the patients’ perception of the quality of services which is consistent with the results of the current study.

Also, a significant relationship was observed between the components of human resources and loyalty. Ayimbillah Atinga et al. (Ayimbillah Atinga, Abekah-Nkrumah, & Ameyaw Domfeh, 2011) considered the lack of opportunity to ask personal questions, limited time spent with physician and the behavior of physician as the main reasons for patient dissatisfaction with hospitals. Arab et al. (Arab et al., 2012) also proved that a strong positive relationship exists between the score of patient loyalty and the dimension of interactive quality (the quality of interaction between staff and patients). The results of these two studies are also consistent with those of the current study.

Despite the significant relationship between CRM and patient loyalty, there is no significant relationship between three components (KM, OI, IT) with patient loyalty. The cause should be sought in objectives and functions of these three components in hospitals. These three components, unlike the other two components (HR, SP), are not tangible and understandable for hospitalized patients, but patients have a direct and understandable relationship with human resources and service-providing processes. Hussain et al. (Hussain et al., 2014) also acknowledged the influence of human resources and the way of providing health services on patients. These three components can be considered as factors supporting and strengthening two components (HR, SP). Also, Kaufman in a report on the impact of internet and information technology on diabetes treatment stated that they can be applied as effective tools for better treatment of more number of patients (Kaufman, 2010). Choi et al. in their study mentioned that there is a significant relationship between the quality of information and the quality of services with consumers’ perceived benefit and satisfaction; Consequently the perceived benefit and satisfaction have considerable impact on individual and organizational performance (Choi et al., 2013). Bahadori et al. also emphasized the importance of organizational structures on the improvement of quality and services for patients’ satisfaction (Bahadori, Yaghoubi, Javadi, & Rahimi, 2015). Indeed, these three components are considered as tools for human resources in hospitals.

## 6. Conclusion

Customer relationship management can be applied to enhance patients’ loyalty; while, two areas of human resources and service-providing process can directly affect patient loyalty, three areas including organizational indicators, information technology and knowledge management are considered as factors influencing patient loyalty to medical wards indirectly.

In order to improve customer relationship management, all its areas should go further in the same direction. In addition, human resources and their functions are considered as the most important dimensions of patient satisfaction and loyalty. It can therefore be concluded that in order to implement CRM, firstly managers should focus on human resources and service-providing processes. However, in long terms, they should know that improvements in these two dimensions requires strong support by knowledge management, information technology and appropriate organizational structures.

**Limitations**

The lack of a hospital in which the CRM has been completely implemented is a limitation of this study, so that each hospital had taken steps in achieving the CRM, so inpatient wards in participating hospitals were selected for the setting of the study.
